# Prevalence of New-Onset Diabetes in Patients Undergoing Pancreatic Surgery and the Association of Glucose Dysregulation With Complications in Pancreatic Cancer

**DOI:** 10.1097/AS9.0000000000000584

**Published:** 2025-06-11

**Authors:** Martyn Stott, Irena Stefanova, Lucy Oldfield, Anthony Evans, James Birch-Ford, Rohith Rao, William Greenhalf, Christopher Halloran, Eithne Costello

**Affiliations:** From the * Department of Molecular and Clinical Cancer Medicine, University of Liverpool, Liverpool, UK; † Department of Pancreatic Surgery, Liverpool University Hospitals Foundation Trust, Liverpool, UK.

**Keywords:** diabetes, new-onset diabetes, pancreatic cancer, pathology, perioperative care

## Abstract

**Objective:**

To determine the prevalence of new-onset diabetes (NOD) in individuals undergoing pancreatic surgery and to explore the implications of glycaemic status on clinicopathological features and outcomes for patients with pancreatic ductal adenocarcinoma (PDAC).

**Introduction:**

PDAC is characterized by a high prevalence of NOD. The prevalence of NOD in individuals undergoing pancreatic surgery for other diseases is less well-documented.

**Methods:**

A retrospective analysis of 483 individuals undergoing pancreatic surgery between 2016 and 2020 was undertaken. For patients with PDAC, associations between glycaemic status and tumor size, cancer stage, grade, postoperative complications, and outcomes were assessed.

**Results:**

Diabetes status was determined for 433 patients. The prevalence of preoperative NOD was higher in PDAC (34.9%; 58/166) compared to ampullary adenocarcinoma (6.3%; 3/48; *P* < 0.001), cholangiocarcinoma (5.6%; 2/36; *P* < 0.001), and intraductal papillary mucinous neoplasms (8.9%; 4/45; *P* = 0.005), but was similar to chronic pancreatitis (30%; 9/30; *P* = 0.909). For 22/58 (37.9%) PDAC patients with NOD, diabetes was undiagnosed until preoperative testing. In individuals undergoing pancreaticoduodenectomy, delayed gastric emptying (DGE) was more frequently associated with glucose dysregulation than with normoglycaemia (32.8% vs 8.3%; *P* = 0.004), while overall postoperative pancreatic fistula (POPF) was less frequently associated with glucose dysregulation than with normoglycaemia (4.7% vs 19.4%; *P* = 0.02).

**Conclusions:**

In contrast to PDAC, NOD was infrequently observed in other pancreatic/periampullary tumors. Of clinical importance, in more than one-third of PDAC patients, NOD was undiagnosed until preoperative assessment. Preoperative glucose dysregulation correlated with an increased rate of DGE and a reduced rate of POPF in pancreaticoduodenectomy.

## INTRODUCTION

Pancreatic ductal adenocarcinoma (PDAC) is the third leading cause of cancer-related deaths in the United States^1^ and the fourth in Europe.^2^ With overall 5-year survival currently at 10%,^3,4^ significant challenges remain for researchers and healthcare professionals working to improve patient outcomes.

The association between PDAC and diabetes mellitus (DM) has attracted attention as an important biological phenomenon and a potential means to detect PDAC earlier.^5^ Approximately 80% of PDAC patients have either DM or impaired fasting blood glucose (FBG) at the time of PDAC diagnosis.^6–8^ When DM occurs, it is most frequently of new-onset, and individuals with new-onset diabetes (NOD) have a 1% probability of being diagnosed with PDAC within 3 years of first meeting the criteria for diabetes.^7,9^ Hyperglycemia begins up to 36 to 30 months before PDAC diagnosis and the duration of prediagnostic hyperglycemia correlates positively with tumor size, as determined by tumor volume.^9^ It is considered unlikely that hyperglycemia is attributable to pancreas gland destruction, as hyperglycemia can begin before PDAC being visible on imaging,^10^ can be associated with very small PDAC tumors,^9^ and can be improved by surgical resection of the cancer.^11^ Understanding the relationship between DM, particularly NOD, and PDAC has the potential to facilitate earlier PDAC detection and improve patient management.

In patients with tumors of the lung, breast, prostate, or bowel, the prevalence of DM is no higher than in noncancer controls.^12^ Whether there may be a stronger relationship between DM and cancers with greater adjacency to the pancreas is not clear. Diabetes has been shown to be associated with ampullary cancer and extrahepatic distal cholangiocarcinoma, however, the prevalence of NOD in these cancer types has, to our knowledge, not been previously reported.^13,14^

The impact of DM on complications after pancreatectomy is not fully understood and studies report variable results.^15–17^ The risk of postoperative pancreatic fistula (POPF) has been reported as reduced^15,16^ and unaffected^17^ by preoperative DM. The frequency of delayed gastric emptying (DGE) following pancreatectomy is reported as comparable between individuals with and without diabetes.^18,19^ In this study, we assessed the prevalence of preoperative diabetes in individuals with PDAC, compared to individuals undergoing pancreatic surgery for other pancreatic/periampullary diseases in a single center in the United Kingdom. We evaluated the extent to which the diagnosis of diabetes was new or known to patients and its impact on postoperative complications and DM treatment at the time of discharge from the hospital.

## METHODS

### Study Design and Setting

A retrospective cohort study of individuals undergoing pancreatic surgery for both benign and malignant diseases at the Liverpool University Hospitals National Health Service (NHS) Foundation Trust, UK, between 2016 and 2020 was undertaken. Individuals were identified from a prospectively maintained database and data gathered from routine contemporaneous input in electronic patient records. Suitability for surgery was assessed following specialist multidisciplinary team meetings for all cases. Postoperative care was according to an institutional standardized enhanced recovery after surgery protocol. Patients were followed up for a minimum of 3 years or until death, whichever occurred first. Research Ethics Committee Approval was obtained from the Institute of Systems, Molecular and Integrative Biology – Research Ethics Committee, University of Liverpool. The study protocol was also approved under the category of service evaluation by the local Development and Innovation Department.

### Definition of Variables

Baseline demographic data, including age, sex, body mass index (kg/m^2^), and operation date, were collected from the local hospital database. Diabetes status was determined at preoperative assessment and on the morning of surgery based on measurement of venous glycated hemoglobin, according to the American Diabetes Association, and recorded diabetes duration based on medical records and patient reports.^20^ Impaired glucose tolerance (IGT) was defined as HbA1c ≥ 42–47 mmol/mol (6.0%–6.4%), diabetes as HbA1c ≥ 48 mmol/mol (6.5%), and normoglycaemia as HbA1c < 42 mmol/mol (6.0%). NOD was defined as diabetes diagnosed within 3 years of surgery, whilst long-standing diabetes mellitus (LSDM) was diabetes of duration greater than 3 years. Diabetes history, including duration of diabetes and treatment, was also recorded to allow the categorization of NOD and LSDM. For malignancy, pathological variables were reported in accordance with the AJCC TMN 8th edition.^21^ Postoperative diabetes care was given by a specialist diabetic team. DGE,^22^ POPF,^23^ and postpancreatectomy haemorrhage^24^ were defined according to the International Study Group in Pancreatic Surgery (ISGPS) definitions. Pancreatic duct size and texture were evaluated by IS and CH, based on preoperative radiological findings, as these parameters were not always recorded in the operation notes. Pancreas texture was categorized as soft (exhibiting fat replacement) or not soft (exhibiting no fat replacement). Chemotherapy data included timing of therapy (neoadjuvant or adjuvant) and treatment regimen. Chemotherapy was determined by a specialist pancreatic medical oncologist and included both standard of care and treatment in a clinical trial setting. Survival data was censored on 3rd May 2024.

### Statistical Analysis

Patients were grouped according to diabetes status, including normoglycaemia, IGT, NOD, and LSDM. The frequency of NOD in different disease groups was compared using the *χ*^2^ test. For patients with PDAC, analyses of clinical characteristics and outcomes according to diabetes state were undertaken. Continuous variables were summarized as median with interquartile range and categorical data were summarized as frequencies of counts with associated percentages. Comparisons across groups were assessed using the Kruskal–Wallis test. The median values of non-normally distributed variables were compared using the Mann–Whitney *U* test. Nominal data were expressed as frequencies and percentages and *χ*^2^ test (or Fisher exact test, when appropriate) applied for comparison. All tests were 2-sided.

Survival was assessed using the Kaplan–Meier approach and comparisons between groups performed using a log-rank test. Multivariable Cox proportional hazard models were used to investigate the impact of multiple factors on the risk of death. A *P* ≤ 0.05 was considered significant.

Analyses were performed using IBM SPSS software (ver.29; SPSS Inc, an IBM company, Chicago, IL). The manuscript was prepared according to STROBE guidelines.

## RESULTS

### Baseline Characteristics—Frequency of NOD in Patients Undergoing Pancreatic Resection

Four hundred and eighty-three individuals (53% male; n = 256/483) underwent pancreatic surgery during the study period (2016 to 2020). The principal nonmalignant indications were intraductal papillary mucinous neoplasms (10.6%; 51/483), chronic pancreatitis (6.8%; 33/483), mucinous cystic neoplasm (3.1%; 15/483), ampullary (2.7%; 13/483), and duodenal adenoma (1.9%; 9/483). The most frequent malignant indications were PDAC (38.3%; 185/483), ampullary adenocarcinoma (10.1%; 49/483), pNET (8.9%; 43/483), and intrapancreatic cholangiocarcinoma (8.3%; 40/483). Definitive diabetes status was available in 433 cases, and these were grouped according to whether individuals had normoglycaemia, IGT, NOD, or LSDM.

Preoperative diabetes status was determined for 89.7% of patients (166/185) with PDAC. More than half (59.0%) had disrupted glucose regulation, including 10.8% (18/166) with IGT, 13.3% (22/166) with LSDM, and 34.9% (58/166) with NOD (Table 1). Of all other disease indications, only chronic pancreatitis exhibited a similar frequency of NOD, with 30% (9/30) of patients in this category (Table 1). Compared to PDAC, the frequency of NOD in other malignancies was low. Significantly fewer patients with ampullary adenocarcinoma (6.3%, 3/48; *P* < 0.001) or intrapancreatic cholangiocarcinoma (5.6%, 2/36; *P* < 0.001) had NOD. Similarly, for individuals with intraductal papillary mucinous neoplasms, the frequency of NOD was significantly lower (8.9%, 4/45; *P* = 0.005) compared to patients with PDAC.

**TABLE 1.** Diabetes Status Based on Pancreatic Histopathological Diagnosis

Histological Diagnosis	NG (N/% of Known)	IGT (N/% of Known)	NOD (N/% of Known)	LSDM (N/% of Known)	Diabetes Status -Known (N/% of Total)	Diabetes Status -Unknown (N/% of Total)
Benign						
Autoimmune pancreatitis	1 (20%)	1 (20%)	2 (40%)	1 (20%)	5 (100%)	0 (0%)
Benign – ampullary tumors	10 (90.9%)	1 (9.1%)	0 (0%)	0 (0%)	11 (84.6%)	2 (15.4%)
Benign – bile duct	3 (75%)	0 (0%)	0 (0%)	1 (25%)	4 (100%)	0 (0%)
Benign – duodenal other	6 (85.7%)	0 (0%)	1 (14.3%)	0 (0%)	7 (87.5%)	1 (12.5%)
Benign – pancreatic cyst	1 (50%)	1 (50%)	0 (0%)	0 (0%)	2 (66.7%)	1 (33.3%)
Benign other	1 (100%)	0 (0%)	0 (0%)	0 (0%)	1 (100%)	0 (0%)
Chronic pancreatitis	12 (40%)	4 (13.3%)	9 (30%)	5 (16.7%)	30 (90.9%)	3 (9.1%)
Duodenal adenoma	7 (77.8%)	0 (0%)	1 (11.1%)	1 (11.1%)	9 (100%)	0 (0%)
IPMN	26 (57.8%)	9 (20%)	4 (8.9%)	6 (13.3%)	45 (88.2%)	6 (11.8%)
Mucinous cystic neoplasm	10 (76.9%)	0 (0%)	2 (15.4%)	1 (7.7%)	13 (86.7%)	2 (13.3%)
Serous cystadenoma	1 (100%)	0 (0%)	0 (0%)	0 (0%)	1 (100%)	0 (0%)
Total	78	16	19	15	128	15
Malignant						
Acinar cell carcinoma	4 (66.7%)	1 (16.7%)	0 (0%)	1 (16.7%)	6 (100%)	0 (0%)
Ampullary adenocarcinoma	35 (72.9%)	4 (8.3%)	3 (6.3%)	6 (12.5%)	48 (98%)	1 (2%)
Cholangiocarcinoma	27 (75%)	4 (11.1%)	2 (5.6%)	3 (8.3%)	36 (90%)	4 (10%)
Duodenal adenocarcinoma	3 (75%)	0 (0%)	0 (0%)	1 (25%)	4 (57.1%)	3 (42.9%)
Lymphoma	1 (100%)	0 (0%)	0 (0%)	0 (0%)	1 (100%)	0 (0%)
Metastasis (other)	5 (83.3%)	1 (16.7%)	0 (0%)	0 (0%)	6 (100%)	0 (0%)
Mucinous adenocarcinoma	1 (50%)	1 (50%)	0 (0%)	0 (0%)	2 (100%)	0 (0%)
PDAC	68 (41%)	18 (10.8%)	58 (34.9%)	22 (13.3%)	166 (89.7%)	19 (10.3%)
pNET	23 (65.7%)	3 (8.6%)	4 (11.4%)	5 (14.3%)	35 (81.4%)	8 (18.6%)
Sarcoma	0 (0%)	1 (100%)	0 (0%)	0 (0%)	1 (100%)	0 (0%)
Total	167	33	67	38	305	35
Whole total	245	49	86	53	433	50

IGT indicates impaired glucose tolerance; IPMN, intraductal papillary mucinous neoplasm; LSDM, long-standing diabetes mellitus; NG, normoglycaemia; NOD, new-onset diabetes; PDAC, pancreatic ductal adenocarcinoma; pNET, pancreatic neuroendocrine tumor.

### Time Interval Between Diabetes Diagnosis and PDAC Diagnosis

For the 58 PDAC patients with NOD, we sought to determine the time interval between NOD diagnosis and the diagnosis of PDAC. For 8.6% (5/58) of patients, diabetes was diagnosed between 2 and 3 years before PDAC diagnosis (Table 2). An additional 24.1% (14/58) of patients received their diabetes diagnosis between 1 and 2 years before PDAC diagnosis. The remaining two-thirds (67.2%; 39/58) of patients were diagnosed with diabetes within 12 months before PDAC diagnosis. Of note, for 37.9% (22/58) of patients with PDAC, there was no evidence of a diagnosis of diabetes before preoperative assessment (Table 2).

**TABLE 2.** Timing of New-Onset Diabetes Diagnosis With Respect to the Date of Surgery in PDAC Patients

Time Between NOD Diagnosis and Date of Pancreatic Surgery	Number of Cases (%)
Diagnosis of NOD at preoperative assessment	22/58 (37.9%)
<1 year	17/58 (29.3%)
1–2 years	14/58 (24.1%)
2–3 years	5/58 (8.6%)

NOD indicates new-onset diabetes; PDAC, pancreatic ductal adenocarcinoma.

### Association of Diabetes Status With Clinicopathological Features in PDAC

We next assessed the association of diabetes status with clinicopathological features of PDAC. Of 185 patients with histologically diagnosed PDAC, those who received neoadjuvant therapy (n = 15) were excluded from analysis. In addition, patients found to have advanced unresectable disease at operation, allowing only for palliative bypass surgery (n = 20), were also excluded. Furthermore, 16 PDAC patients with unknown diabetes status were not considered for analysis.

Diabetes status (normoglycaemia, IGT, NOD, and LSDM) did not significantly associate with clinicopathological characteristics such as tumor differentiation, TNM stage, resection margin, lymphovascular and perineural invasion, and maximum tumor diameter (Table 3). Furthermore, there was no significant difference in median FBG levels or median HbA1c between well-, moderately-, and poorly differentiated tumors (*P* = 0.655, and *P* = 0.728, respectively).

**TABLE 3.** Impact of Diabetes Status on the Clinicopathological Features of PDAC

	Normoglycaemia (n = 53)	IGT (n = 16)	NOD (n = 46)	LSDM (n = 19)	*P*
Age (years)	68 (59–75)	67 (65–78)	71 (64–77)	70 (61–75)	0.543
Sex–Male	21/53 (39.6%)	7/16 (43.8%)	29/46 (63%)	8/19 (42.1%)	0.110
BMI (kg/m^2^)	25 (23–28)	26 (24–27)	25 (23–27)	25 (23–28)	0.964
Smoking status					0.649
Yes	13/53 (24.5%)	4/16 (25%)	5/44 (11.4%)	5/19 (26.3%)	
No	23/53 (43.4%)	7/16 (43.8%)	26/44 (59.1%)	9/19 (47.4%)	
Ex-smoker	17/53 (32.1%)	5/16 (31.3%)	13/44 (29.5%)	5/19 (26.3%)	
Operation performed					0.611
Left pancreatectomy	10/53 (18.9%)	2/16 (12.5%)	7/46 (15.2%)	3/19 (15.8%)	
PPPD	35/53 (66%)	11/16 (68.8%)	36/46 (78.3%)	16/19 (84.2%)	
Kausch–Whipple	1/53 (1.9%)	1/16 (6.3%)	1/46 (2.2%)	0/19 (0%)	
Total pancreatectomy	7/53 (13.2%)	2/16 (12.5%)	2/46 (4.3%)	0/19 (0%)	
Tumor differentiation					0.617
Well	0/53 (0%)	0/16 (0%)	0/46 (0%)	1/19 (5.3%)	
Moderate	31/53 (58.5%)	9/16 (56.3%)	24/46 (52.2%)	10/19 (52.6%)	
Poor	24/53 (39.6%)	7/16 (43.8%)	21/46 (45.7%)	8/19 (42.1%)	
Undifferentiated	1/53 (1.9%)	0/16 (0%)	1/46 (2.2%)	0/19 (0%)	
T stage					0.727
T1	4/53 (7.5%)	3/16 (18.8%)	3/46 (6.5%)	0 (0%)	
T2	15/53 (28.3%)	3/16 (18.8%)	15/46 (32.6%)	7/19 (36.8%)	
T3	33/53 (62.3%)	10/16 (62.5%)	27/46 (58.7%)	12/19 (63.2%)	
T4	1/53 (1.9%)	0/16 (0%)	1/46 (2.2%)	0/19 (0%)	
N stage					0.324
N0	5/53 (9.4%)	5/16 (31.3%)	12/46 (26.1%)	4/19 (21.1%)	
N1	36/53 (67.9%)	9/16 (56.3%)	26/46 (56.5%)	10/19 (52.6%)	
N2	12/53 (22.6%)	2/16 (12.5%)	8/46 (17.4%)	5/19 (26.3%)	
Perineural invasion	51/53 (96.2%)	16/16 (100%)	43/46 (93.5%)	19/19 (100.0%)	0.502
Lymphovascular invasion	47/53 (88.7%)	13/16 (81.3%)	42/46 (91.3%)	18/19 (94.7%)	0.589
Max tumor size (mm)	31 (25–42)	30 (25–40.8)	33 (27.5–42.5)	30 (27–40)	0.864
Resection margin positive (R_1_)	40/53 (75.5%)	12/16 (75%)	35/46 (76.1%)	11/19 (57.9%)	0.453

Continuous data were compared using Kruskal–Wallis tests. Categorical data was compared using *χ*^2^. *P* < 0.05 was considered significant.

BMI indicates body mass index; IGT, impaired glucose tolerance; KW-PD, Kausch–Whipple pancreaticoduodenectomy; LSDM, long-standing diabetes mellitus; NOD, new-onset diabetes; PPPD, pylorus-preserving pancreaticoduodenectomy.

Additionally, no correlation was found between maximum tumor diameter and preoperative FBG levels, or HBA1c levels [*P* = 0.861, Spearman’s rho = 0.015, 95% confidence interval (CI) = (−0.157–0.186); *P* = 0.730, rho = 0.031, 95% CI = (−0.149–0.208), respectively].

### Association of Glucose Dysregulation With Complications and Clinical Outcomes of Resectable PDAC

One hundred and fifty patients underwent pancreatic resection for PDAC, including pylorus-preserving pancreaticoduodenectomy (70.7%, 106/150), Kausch–Whipple pancreaticoduodenectomy (5.3%, 8/150), left pancreatectomy (16%, 24/150), and total pancreatectomy (8%, 12/150). Individuals undergoing total pancreatectomy were considered in the analysis of DGE but excluded from POPF analysis. Incidences of postoperative complications following pancreaticoduodenectomy (Kausch–Whipple pancreaticoduodenectomy and pylorus-preserving pancreaticoduodenectomy) and left pancreatectomy in normoglycaemia and glucose dysregulation are shown in Table 4. Two incidences of postpancreatectomy hemorrhage occurred, 1 grade A in an individual with NOD and 1 grade B in an individual with normoglycaemia. The most frequently observed postoperative complications were Clavien-Dindo grade 2 and 3a, 30.2% (45/149) and 14.8% (22/149), respectively. Additionally, grade 1 complications were 3.4% (5/149), grade 3b – 0.7% (1/149), grade 4 – 0.7% (1/149), and grade 5 – 0.7% (1/149).

**TABLE 4.** Incidence of Postoperative Complications Based on Operation Type and Glucose Regulation

	Pancreaticoduodenectomy (KW-PD + PPPD) N = 114 (Missing Values = 14)			Left Pancreatectomy N = 24 (Missing Values = 2)		
Glucose Regulation Status	Normoglycaemia (N =36)	Glucose Dysregulation (IGT + NOD + LSDM) (N = 64)		Normoglycaemia (N = 10)	Glucose Dysregulation (IGT + NOD + LSDM) (N = 12)	
DGE rate*	8.3% (3/36)	32.8% (21/64)	***P* = 0.004**	10% (1/10)	16.7% (2/12)	*P* = 0.571
DGE grade			***P* = 0.052**			*P* = 0.645
A	2.8% (1/36)	7.8% (5/64)		10% (1/10)	8.3% (1/12)	
B	5.6% (2/36)	18.8% (12/64)		0% (0/10)	8.3% (1/12)	
C	0% (0/36)	6.3% (4/64)		0% (0/10)	0% (0/12)	
Overall POPF rate	19.4% (7/36)	4.7% (3/64)	***P* = 0.024**	40% (4/10)	25% (3/12)	*P* = 0.384
POPF grade			***P* = 0.050**			*P* = 0.813
BL	8.3% (3/36)	3.1% (2/64)		10% (1/10)	8.3% (1/12)	
B	11.1% (4/36)	1.6% (1/64)		30% (3/10)	16.7% (2/12)	
C	0% (0/36)	0% (0/64)		0% (0/10)	0% (0/12)	
PD size			***P* = 0.026**			*P* = 0.135
<3	44.4% (16/36)	23.4% (15/64)		30% (3/10)	63.6% (7/11/)	
>3	55.6% (20/36)	76.6% (49/64)		70% (7/10)	36.4% (4/11)	
Soft pancreas texture			*P* = 0.336			*P* = 0.608
No	75% (27/36)	68.8% (44/64)		60% (6/10)	63.6% (7/11)	
Yes	25% (9/36)	31.3% (20/64)		40% (4/10)	36.4% (4/11)	
PPH rate	2.8% (1/36)	1.6% (1/64)	*P* = 0.593	0% (0/10)	0% (0/12)	–
PPH grade			*P* = 0.607			–
A	0% (0/36)	1.6% (1/64)		0% (0/10)	0% (0/12)	
B	2.8% (1/36)	0% (0/64)		0% (0/10)	0% (0/12)	
C	0% (0/36)	0% (0/64)		0% (0/10)	0% (0/12)	

*P* values where *P* is less than or approaches 0.05 are highlighted in bold.

* Note the DGE rate in patients who underwent total pancreatectomy (N = 11, 1 missing diabetes status) was not included in this table; however it was analyzed: overall rate of 20% (2/10 patients).

BL indicates biochemical leak, formally type A POPF; DGE, delayed gastric emptying; IGT, impaired glucose tolerance; KW-PD, Kausch–Whipple pancreatoduodenectomy; LSDM, long-standing diabetes mellitus; NOD, new-onset diabetes; PD, Pancreatic duct; POPF, postoperative pancreatic fistula; PPH, postpancreatectomy hemorrhage; PPPD, pylorus-preserving pancreaticoduodenectomy.

For the analysis of the effects of glucose dysregulation on DGE, 150 patients were identified, with the subsequent exclusion of 18, due to one early postoperative death and 17 individuals with unknown diabetes status. A total of 132 patients were included in the final analysis (pancreaticoduodenectomy n = 100; left pancreatectomy n = 22, total pancreatectomy n = 10). Grouping all operation types together, the DGE rate was higher in individuals with glucose dysregulation (28.7%; 24/80) compared to normoglycaemia (9.6%; 5/52; *P* = 0.009). Analysis of the rate of DGE by operation type revealed that in the pancreaticoduodenectomy group, 24% (24/100) experienced DGE of which one-third (32.8%; 21/64) of patients had glucose dysregulation (IGT, NOD, and LSDM) compared to 8.3% (3/36) in the normoglycaemic group (Fisher exact; *P* = 0.004). In comparison, the left pancreatectomy group showed an overall DGE rate of 13.6% (3/22) with no significant difference in DGE occurrence in glucose dysregulation versus normoglycaemia (10%; 1/10 vs 16.7%; 2/12, respectively, *P* = 0.571; Table 4). The incidence of DGE in the total pancreatectomy group was 20% (2/10), including 25% (1/4) in glucose dysregulation versus 16.7% (1/6) in normoglycaemia (*P* = 0.667, data not presented). Furthermore, glucose dysregulation was associated with significantly increased risk of DGE independently of age, sex, BMI, presence of POPF, and operation type [odds ratio (OR) = 5.42, CI = 95% (1.73–16.98), *P* = 0.004] (Table 5).

**TABLE 5.** Multivariable Logistic Regression Assessing the Association Between Glucose Regulation Status and DGE and POPF in Patients Undergoing Resection for PDAC

	OR	95% CI	*P*
DGE			
Glucose dysregulation	5.42	1.73–16.98	**0.004**
Sex–Male	0.58	0.23–1.47	0.247
Age	0.96	0.92–1.01	0.107
BMI	1.01	0.90–1.13	0.905
POPF	0.95	0.09–10.5	0.967
Pancreaticoduodenectomy	2.84	0.59–13.64	0.192
POPF			
Normoglycaemia	3.54	1.09–11.49	**0.035**
Sex–Male	1.33	0.38–4.59	0.653
Age	0.99	0.94–1.06	0.960
BMI	1.13	0.98–1.29	0.096
Pancreaticodudenectomy	0.21	0.06–0.74	**0.015**
Soft pancreas texture	0.68	0.18–2.56	0.570
Duct size >3 mm	0.30	0.09–1.07	0.064

*P* values where *P* is less than 0.05 are highlighted in bold.

BMI indicates body mass index; DGE, delayed gastric emptying; PDAC, pancreatic ductal adenocarcinoma.

For the analysis of POPF, of 150 patients, 28 were excluded (12 having undergone total pancreatectomy and 16 with unknown diabetes status). A total of 122 individuals were included in the final analysis (pancreaticoduodenectomy n = 100; left pancreatectomy n = 22). The overall POPF rate across both operation types was 13.9% (17/122), with significantly increased POPF occurrence in normoglycaemic patients (23.9%; 11/46) in comparison to those with glucose dysregulation (7.9%; 6/76; Fisher exact *P* = 0.015). In a subset analysis of pancreaticoduodenectomy patients, the overall incidence of POPF was 10% (10/100) with this complication continuing to occur more often in normoglycaemia (19.4%; 7/36) versus glucose dysregulation (4.7%; 3/64), Fisher exact, *P* = 0.024. Of note, pancreatic duct size >3 mm was more frequently observed in the glucose dysregulation group than in normoglycaemia (76.6%, 49/64 vs 55.6%, 20/36; *P* = 0.026). In the left pancreatectomy group, the overall POPF rate was 31.9% (7/22) including 40% (4/10) in normoglycaemia versus 25% (3/12) in glucose dysregulation (*P* = 0.384). Of note, the indicated protective association of glucose dysregulation with POPF was particularly pronounced in patients with NOD, where we recorded no cases (0/43) of POPF. The increased risk of POPF inferred by normoglycaemia was independent of age, sex, BMI, operation type, pancreas texture, and duct size [OR = 3.54, 95% CI = (1.09–11.49), *P* = 0.035]; Table 5). Pancreatico-jejunostomy technique was not included as a covariate in this analysis, as information on the type of anastomosis was unavailable due to assessor blinding within the PANASTA trial.^25^

## ASSOCIATION BETWEEN GLUCOSE DYSREGULATION AND SURVIVAL

**FIGURE 1.**
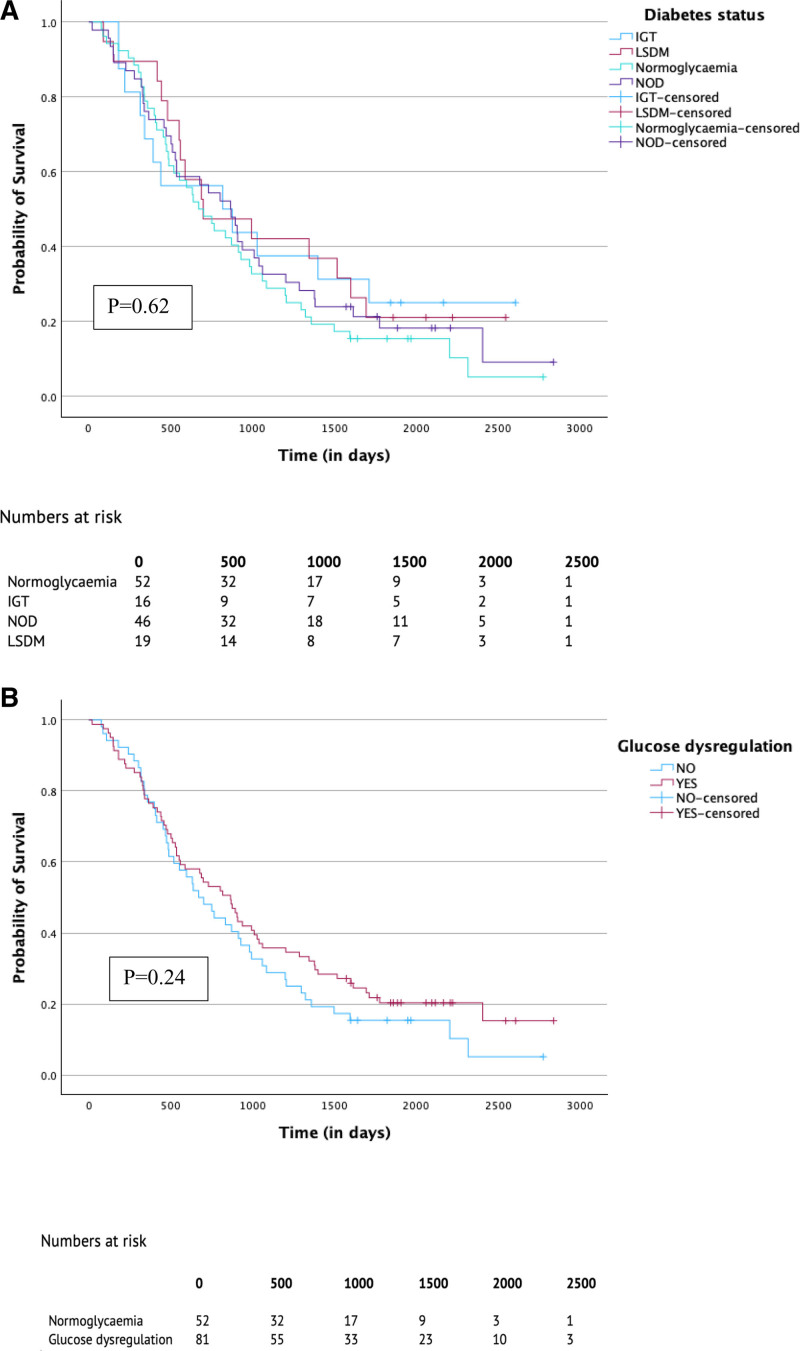
Kaplan–Meier estimates of overall survival of PDAC patients according to Diabetes status (A) and Glucose dysregulation (B). DM indicates diabetes mellitus; IGT, impaired glucose tolerance; LSDM, long-standing diabetes mellitus; NOD, new-onset diabetes.

Median overall survival in patients with PDAC was 2.1 years (interquartile range 1.6–2.6 years; Fig. 1A). No significant difference in median overall survival was found between individuals with glucose dysregulation compared to those with normoglycaemia [2.4 years (95% CI = 1.81–2.99) vs 1.9 years (95% CI = 1.30–2.42), respectively; *P* = 0.239; Fig. 1B]. Overall survival was significantly associated with higher tumor stage T4 [hazard ratio (HR) = 67.59, 95% CI = (2.47–1848.90), *P* = 0.013] and higher nodal stage N2 [HR = 2.37, 95% CI = (1.02–5.52), *P* = 0.045], poor tumor differentiation [HR = 1.64, 95% CI = (1.04–2.58), *P* = 0.034] and adjuvant chemotherapy [HR = 0.31, 95% CI = (0.14–0.70), *P* = 0.005], whilst glucose dysregulation did not significantly associate with survival [HR = 0.83, 95% CI = (0.53–1.29), *P* = 0.411] (Table 6).

**TABLE 6.** Multivariate Cox Regression Analysis of Factors Influencing Survival

	HR	95% CI	*P*
Diabetes state			
Normoglycaemia	Ref		
Glucose dysregulation	0.83	0.53–1.29	0.411
T stage			
T1	Ref		
T2	9.08	0.79–103.49	0.076
T3	13.67	1.23–151.93	0.033
T4	67.59	2.47–1848.90	0.013
N stage			
N0	Ref		
N1	1.47	0.68–3.18	0.331
N2	2.37	1.02–5.52	0.045
R status			
R0	Ref		
R1	0.87	0.49–1.56	0.648
Differentiation			
Moderate	Ref		
Poor	1.64	1.04–2.58	0.034
Perineural invasion			
No	Ref		
Yes	1.13	0.10–12.47	0.921
Lymphovascular invasion			
No	Ref		
Yes	0.94	0.39–2.23	0.890
Adjuvant therapy			
No	Ref		
Yes	0.31	0.14–0.70	0.005

### Change in Antidiabetes Medications Post-PDAC Resection

Preoperative and postoperative diabetes medication (both oral and insulin) history was available for 122 PDAC patients. All PDAC patients undergoing total pancreatectomy were discharged on insulin. Preoperative and immediate postoperative diabetes management varied amongst diabetes states. For patients without preoperative diabetes (IGT and normoglycaemia, n = 63), 33.3% (21/63) were discharged with diabetes therapy, including oral antidiabetic medications and insulin, after surgery. Additionally, patients with IGT received postoperative antidiabetes therapy more frequently compared to normoglycaemic patients, 20% (3/15) versus 12.2% (6/49), respectively. Of those with LSDM, 85.7% (18/21) were on antidiabetic medications preoperatively, whereas postoperatively all individuals were receiving treatment (*P* < 0.001), including increased insulin requirements from 52.4% (11/21) before resection to 85.7% (18/21) after surgery (*P* = 0.008). In the NOD group, 40.5% (15/37) of patients were receiving treatment for diabetes preoperatively. This increased to 77.8% (25/37) postoperatively (*P* < 0.001). The use of insulin in the NOD group increased from 21.6% (8/37) before surgery to 41.3% (19/37) on discharge (*P* < 0.001).

## DISCUSSION

Determining how a new diagnosis of diabetes in individuals over 50 years is best incorporated into strategies for early PDAC detection, and how diabetes optimization perioperatively could affect outcomes is vitally important.^26,27^ However, the reported prevalence of diabetes in PDAC studies varies, with a diversity of diagnostic criteria employed, including FBG, HbA1c, RBG (random blood glucose), and self-reporting. To minimize reliance on self-reporting of diabetes or on the extrapolation of diabetes status based on antidiabetic medication use, both of which can lead to an underestimation of diabetes,^28–30^ we measured HbA1c at preoperative assessment. Our study found diabetes in 48.2% (80/166) of patients undergoing surgery for PDAC. Diabetes was most frequently diagnosed within 3 years of the date of PDAC surgery (73%; 58/80), with 67% of diagnoses made in the 12 months before PDAC surgery.

The high prevalence of NOD in our cohort was uniquely associated with PDAC, when compared to other pancreatic malignancies (6.1% in ampullary adenocarcinoma and 5% in distal cholangiocarcinoma). The literature is scarce on the prevalence of NOD broken down by type of hepatobiliary cancer. However, diabetes of undefined duration has been reported to occur in 12% (32/266) and in 22.5% (428/1904) of patients undergoing pancreaticoduodenectomy for ampullary adenocarcinoma^31^ or cholangiocarcinoma,^14^ respectively.

Of clinical importance, our results revealed that in over one-third of individuals (37.9%), diabetes had gone undiagnosed until HbA1c measurement was undertaken preoperatively. The 2019 UK National Screening Committee review recommended against population-based screening for T2DM based on lack of evidence that early diagnosis by screening will be of greater benefit than the current NHS programmes for diabetes.^32^ The UK NHS Health Check includes measurements of height, weight, waist, blood pressure and cholesterol, and incorporates a diabetes risk assessment which can trigger investigations for diabetes. People between the ages of 40 and 74 are invited to attend an NHS Health Check every 5 years.^33^ The lack of a yearly diabetes risk assessment, combined with HbA1c testing only in those with a high risk score, and the exclusion of individuals over the age of 74, likely contributes to the large proportion of PDAC patients whose diabetes goes undetected until presurgical assessment. Addressing these deficits to improve community-based diabetes screening may lead to an earlier diagnosis of diabetes for PDAC patients, which may in-turn facilitate early cancer detection strategies.^26,27^

In our study, DM status did not significantly associate with clinicopathological characteristics, such as tumor size (diameter) or differentiation status. This finding aligns with a recent study which subcategorized 2643 patients undergoing surgery for PDAC into 3 groups – symptomatic, asymptomatic and patients with NOD, and reported no differences in pathological characteristics amongst the 3 groups.^34^ In contrast, a study of 488 PDAC patients reported that, independently of age, BMI and tumor grade, diabetic subjects had larger tumors (determined by volume) than nondiabetic subjects.^35^ Furthermore, smaller poorly differentiated PDAC tumors (1.5cc) have been shown to be associated with the same levels of FBG compared to larger well- and moderately differentiated tumors (5.8cc).^9^

DGE is one of the most frequent complications following pancreatic resection. Its incidence ranges from 17% to 70%, due to inconsistencies in historical definitions before development of the widely accepted ISGPS criteria.^23,36^ We observed DGE to be more frequent in patients with glucose dysregulation compared to those with normoglycaemia. Furthermore, there is an established association between diabetes and gastroparesis with 50% of diabetic patients experiencing DGE in cases of suboptimal glycaemic control.^37,38^ However, the literature is inconsistent regarding the impact of preoperative diabetes on the development of DGE post-PDAC resection. A number of studies have reported no significant difference in DGE incidence amongst diabetic and nondiabetic PDAC patients undergoing surgery.^18,39,40^ On the contrary, in a meta-analysis by Qu et al,^41^. preoperative diabetes predicted an increased risk of DGE (OR 1.49, 95% CI = 1.03–2.17), with Cai et al^36^ further reporting diabetes as a risk factor for severe DGE (HR = 3.00, 95% CI = 1.03–8.78; *P* = 0.045). Our results are in alignment with these latter findings. Additional studies are required to determine if preoperative optimization of diabetes management can reduce the development of DGE.

The association between preoperative DM and POPF, has also been evaluated in several studies, again with equivocal results. A multicenter study of 1898 pancreatoduodenectomy patients (35.2% with PDAC), reported that independent risk factors for clinically relevant (CR) POPF included higher preoperative BMI and soft pancreatic texture. However, preoperative diabetes was not found to predict the rate of POPF.^42^ In a study that focused solely on PDAC (n = 174 patients), again preoperative DM was not independently associated with POPF [HR = 1.01; 95% CI = (0.50–2.07); *P* = 0.97].^17^ Other studies have reported comparable findings.^43,44^ Here, we observed the POPF rate to be lower amongst patients with glucose dysregulation compared to those who were normoglycaemic. Consistent with this, Malleo et al^15^, demonstrated a greater incidence of CR-POPF amongst individuals without diabetes, who were more likely to have a soft-textured pancreas gland. Malleo further showed DM to be a negative predictor of CR-POPF, independent of pancreas texture, size of pancreatic duct, and gender.

Mathur et al^16^ hypothesized that individuals developing POPF have increased pancreatic fat. In a case-matched analysis the authors found that patients with fistula (n = 40) were less likely to have diabetes compared to patients without POPF (n = 40; 13% vs 33%, respectively; *P* < 0.005). Moreover individuals with POPF had increased intralobular (*P* < 0.001), interlobular (*P* < 0.05), and total pancreatic fat (*P* < 0.001), and less fibrosis (*P* < 0.001).^16^ Although other studies have found BMI to be an independent positive predictor for POPF,^42,43^ Mathur and colleagues did not observe large differences in BMI between the 2 comparison groups (fistula and control).^16^ Larger studies examining the factors leading to increase in pancreatic fat, its association with BMI, and its correlation to POPF incidence are required.

We observed no significant difference in overall survival in PDAC cases with and without glucose dysregulation. Other studies have reported similar findings in 5-year survival between diabetic and nondiabetic subjects undergoing pancreaticoduodenectomy.^28,45^ However, NOD has been reported to be associated with poorer over survival compared to non-DM,^46^ and diabetes of all duration has been reported to be associated with reduced survival following PDAC resection and adjuvant chemotherapy.^30^ By contrast, Honselmann et al^34^ reported a significantly higher 5-year survival in patients with recent-onset diabetes (58%) compared to those without (28%), (*P* = 0.013) aligning with other studies showing improved overall survival in PDAC cases with diabetes.^40,47^

Finally, we observed that for PDAC patients with NOD, the percentage receiving treatment for diabetes, including both oral antidiabetic medications and insulin, increased from 40.5% preoperatively to 77.8% at discharge from the hospital, with insulin use increase from 21.6% to 41.3%. DM status was not followed up beyond the point of patient discharge due to limited availability of data. Interestingly, Pannala et al^8^, have previously reported preoperative diabetes of < 2 years duration resolving in 57% (17/30) individuals who were followed up for a median of 48 days postpancreatic resection for PDAC.

Limitations of this study include its retrospective design and the fact it was conducted from a single center. Although our sample size was relatively small potentially affecting the power of our study and limiting the generalisability of our findings, the biochemical criteria for diabetes diagnosis in the preoperative period were accurately captured. Due to a lack of access to a radiomics-based tool for the evaluation of preoperative fistula-risk score, pancreatic duct size and pancreatic texture were assessed by 2 pancreatic surgeons based on preoperative radiological features. Oncological services were also centralized, which allowed for consistent collection of data on chemotherapy and survival. Future studies should confirm these findings in a prospective, multicenter setting and would benefit from detailed collection of pre- and post-operative diabetes treatment data.

In conclusion, we found a high frequency of NOD uniquely in individuals undergoing surgery for PDAC compared to other pancreatic malignancies and observed effects on the rate of complications of surgery. The fact that over one-third of PDAC patients received their diagnosis of diabetes at preoperative assessment has implications for future detection strategies based on using NOD as a warning sign of pancreatic cancer. A greater understanding of the pathophysiology of PDAC-related diabetes is urgently needed to improve the options available for both earlier detection and treatment of this disease.
